# Platelet-to-C-reactive protein ratio stratifies surgical risk and mortality in necrotizing enterocolitis neonates with portal venous gas

**DOI:** 10.3389/fped.2025.1686076

**Published:** 2026-01-02

**Authors:** Xinyin Zhang, Huan Wei, Qi Tan, Zhengli Wang, Zhenhua Guo, Wei Liu, Jian Cao

**Affiliations:** 1 Department of Neonatology, Children’s Hospital of Chongqing Medical University, National Clinical Research Center for Child Health and Disorders, Ministry of Education Key Laboratory of Child Development and Disorders, Chongqing Key Laboratory of Structural Birth Defect and Reconstruction, Chongqing, China; 2 Department of General & Neonatal Surgery, Children’s Hospital of Chongqing Medical University, National Clinical Research Center for Child Health and Disorders, Ministry of Education Key Laboratory of Child Development and Disorders, Chongqing Key Laboratory of Child Neurodevelopment and Cognitive Disorders, Chongqing, China

**Keywords:** necrotizing enterocolitis, portal venous gas, platelet-to-C-reactive protein ratio, surgical intervention, mortality

## Abstract

**Background:**

Portal venous gas (PVG) represents a severe complication of necrotizing enterocolitis (NEC), typically signaling disease progression and a poor prognosis. While PVG has traditionally been regarded as an indication for surgical intervention in NEC, accumulating clinical evidence indicates that not all cases require operative management. Currently, surgical decision-making for NEC neonates with PVG primarily relies on subjective clinical assessments by physicians, resulting in substantial management uncertainty. The platelet-to-C-reactive protein ratio (PCR), an objective and convenient biomarker integrating information on disseminated intravascular coagulation and systemic inflammation, has demonstrated predictive value in neonatal sepsis and other conditions. However, the utility of the PCR has not yet been explored in NEC-PVG patients. This study retrospectively evaluates the predictive value of the PCR for surgical intervention and prognosis in this high-risk population, aiming to facilitate early identification and improve clinical outcomes.

**Methods:**

This retrospective single-center cohort study analyzed 186 neonates diagnosed with NEC (Bell stage ≥ IIa) and ultrasonographically confirmed PVG (2021–2024). We evaluated the PCR, calculated at the time of PVG diagnosis, for predicting surgical intervention and 30-day mortality by employing multivariate logistic regression (which included collinearity assessment and bootstrap validation) to identify independent factors and receiver operating characteristic (ROC) analysis to assess predictive performance and determine optimal cutoff values, supplemented by sensitivity analyses.

**Results:**

The cohort demonstrated a surgical intervention rate of 37.10% (69/186) and a 30-day mortality of 13.98% (26/186). Multivariate analysis identified the PCR as an independent predictor for both surgical intervention (adjusted odds ratio (aOR) = 0.77, 95% CI: 0.69–0.86) and mortality (aOR = 0.80, 95% CI: 0.69–0.92). ROC curve analysis established PCR thresholds of ≤4.84 × 10^9^/mg for surgical intervention (AUC 0.84; sensitivity 80%, specificity 85%) and ≤2.57 × 10^9^/mg for mortality prediction (AUC 0.80; sensitivity 69%, specificity 91%).

**Conclusions:**

This study suggests that the PCR is a promising predictor of surgical intervention and mortality risk in NEC-PVG patients, demonstrating superior predictive performance compared to individual parameters. Based on these findings, we propose the incorporation of PCR testing into standard clinical monitoring protocols, using 4.84 × 10^9^/mg as a potential cutoff value to inform therapeutic decision-making. This approach may improve the clinical management and outcomes in this high-risk population.

## Introduction

Neonatal necrotizing enterocolitis (NEC) stands as the gravest gastrointestinal condition affecting newborns, showing particular prevalence among premature and underweight infants, where mortality rates reach 20%–30% (1, 2). While modern medicine has made strides in intensive care and surgical management, this condition persists as a primary contributor to newborn deaths and serious health consequences (3, 4). Among the serious complications of this disease, portal venous gas (PVG) has drawn considerable attention due to its significant clinical implications.

PVG is a distinctive radiological finding of NEC, resulting from disruption of the intestinal mucosal barrier, bacterial translocation, and subsequent portal venous gas embolism formation (5, 6). Many studies have shown that NEC infants with PVG exhibit poorer prognoses, with significantly elevated mortality rates (40%–50%) compared to those without (7–10). As a result, PVG itself has been traditionally regarded as both an indicator of disease progression and a compelling indication for exploratory laparotomy. Clinical practice, however, presents a more complex picture: not all PVG cases need immediate surgical intervention, as a subset of patients achieve favorable outcomes through conservative management. This therapeutic paradox—where traditional surgical criteria conflict with observed clinical trajectories—creates substantial management ambiguity, highlighting a critical dilemma in neonatal surgical practice: the urgent need to establish reliable parameters for accurately assessing disease severity and determining optimal surgical timing in PVG-associated NEC cases.

However, the current decision-making process for surgical intervention in NEC patients with PVG remains primarily dependent on clinical experience and subjective assessment. Although the Bell staging criteria are conventionally employed to evaluate disease severity and establish operative indications, significant interobserver variability exists in their interpretation (11), which reduces predictive accuracy in neonates with PVG. This underscores the need for more objective evaluation parameters.

In recent years, multiple pathophysiological indicators have been combined to enhance the accuracy of predictions. The platelet-to-C-reactive protein ratio (PCR), a composite biomarker simultaneously evaluating coagulation function and inflammatory status, has demonstrated superior predictive performance of prognoses compared to isolated platelet or C-reactive protein measurements in sepsis, chronic heart failure, and COVID-19 (12–14), offering new insights for surgical decision-making and prognosis prediction in NEC patients with PVG. Nevertheless, systematic investigations evaluating the predictive utility of the PCR in NEC, particularly in the high-risk PVG subgroup, remain notably lacking.

This study aims to evaluate the predictive value of the PCR for determining surgical intervention requirements and prognostic stratification in NEC patients with PVG through a retrospective analysis. We hypothesize that the PCR, as a composite biomarker, will demonstrate superior accuracy compared to individual parameters in identifying high-risk cases requiring surgical management and those with poorer prognoses, thereby potentially providing more reliable objective evidence to guide clinical decision-making.

## Materials and methods

This single-center, retrospective cohort study was conducted in the neonatal intensive care unit (NICU) of the Children's Hospital of Chongqing Medical University, which functions as a tertiary referral center and national medical center for children in China. The study period ranged between June 2021 and August 2024. The study protocol was approved by the Institutional Review Board of our institution (Approval No. CHMU2021-189) and was conducted in accordance with the ethical principles of the Declaration of Helsinki. As a non-interventional retrospective study utilizing anonymized data with minimal risk to participants, the ethics committee waived the requirement for informed consent. No clinical trial registration was required, given the observational design of the study.

### Study population and design

This retrospective study enrolled 186 neonates with NEC and ultrasonographically confirmed PVG who were consecutively admitted to the NICU at Chongqing Children's Hospital from June 2021 to August 2024. Inclusion criteria comprised (1) Bell stage ≥ IIa NEC, (2) PVG independently verified by two experienced pediatric sonographers, and (3) availability of complete laboratory parameters at the time of PVG diagnosis. We excluded patients with congenital gastrointestinal malformations (e.g., meconium peritonitis or Hirschsprung's disease), those with hematologic disorders, those receiving platelet-altering medications or transfusions, and cases with incomplete data.

### Data collection

All data were extracted from electronic medical records, including the following: (1) demographic characteristics (sex, delivery mode, APGAR scores, corrected gestational age, and weight at PVG diagnosis); (2) maternal and perinatal information (maternal age, pregnancy status, complications including hypertensive disorders, diabetes, cholestasis, and hypothyroidism, and use of antenatal steroids/antibiotics); (3) pre-NEC clinical information (transfusion history, congenital heart disease, bronchopulmonary dysplasia, exclusive breastfeeding, and antibiotic use); (4) clinical manifestations (fever, vomiting, abdominal distension, diarrhea, bloody stools, and decreased bowel sounds); (5) laboratory tests (complete blood count parameters and C-reactive protein at PVG diagnosis); and (6) imaging findings (abdominal and cardiac ultrasound results). The PCR at PVG diagnosis served as the primary exposure. The primary outcome was surgical intervention with histopathologically confirmed intestinal necrosis, while 30-day mortality constituted the secondary outcome. Two researchers independently collected all data with cross-verification.

### Definition

PVG was defined as the detection of mobile hyperechoic dots or linear streaks within the portal venous branches or main trunk, accompanied by characteristic hemodynamic changes, as identified by experienced sonographers using standardized ultrasound systems (Philips iU 22 or Aixplorer, SuperSonic Imagine, Aix-en-Provence, France) and confirmed through PACS workstation review. All imaging interpretations were performed independently by board-certified pediatric ultrasound physicians with consensus documentation.

The PCR was defined as the absolute platelet count (×10^9^/L) divided by the CRP level (mg/L), with both parameters measured from the same venous blood samples collected within 30 min following confirmation of PVG. To ensure sample integrity, all laboratory analyses were completed within 1 h of blood collection. Platelet counts were measured using a Sysmex XN-9000 hematology analyzer, with CRP levels determined by immunoturbidimetry (Roche Cobas c502 analyzer).

Surgical intervention was defined as either exploratory laparotomy or peritoneal drain placement. Indications included (1) radiographically confirmed pneumoperitoneum and (2) clinical deterioration following maximal medical management (manifested by persistent abdominal tenderness/erythema/discoloration, refractory metabolic acidosis, or hemodynamic instability). All cases required consensus evaluation by at least two board-certified pediatric surgeons and consulting neonatologists.

### Statistics

All statistical analyses were performed using SPSS (version 26; IBM Corp., Chicago, IL, USA) and R (version 4.3.1; R Foundation for Statistical Computing, Vienna, Austria). Continuous variables are reported as mean ± SD (analyzed by the *t*-test) or median [IQR] (analyzed by the Mann–Whitney *U*-test); categorical variables are reported as *n* (%) (analyzed by the *χ*^2^/Fisher's exact test). Variables with *P* < 0.05 in univariate analysis and clinically relevant factors were selected as candidates for multivariable logistic regression models via the enter method. Prior to modeling, multicollinearity was assessed using the variance inflation factor (VIF), and variables with a VIF < 5 were retained. Model fit was evaluated using the Hosmer–Lemeshow test and internally validated via bootstrap resampling (2,000 replicates). Adjusted associations are reported as odds ratios, with *P* < 0.05 considered statistically significant. The predictive performance of independent hematological risk factors was assessed by receiver operating characteristic (ROC) curve analysis, with optimal cutoff values determined by maximizing the Youden index. The robustness of the predictive performance and cutoff stability were further evaluated by sensitivity analyses across key subgroups.

## Results

### Patients' baseline characteristics and clinical information

**Figure 1:**
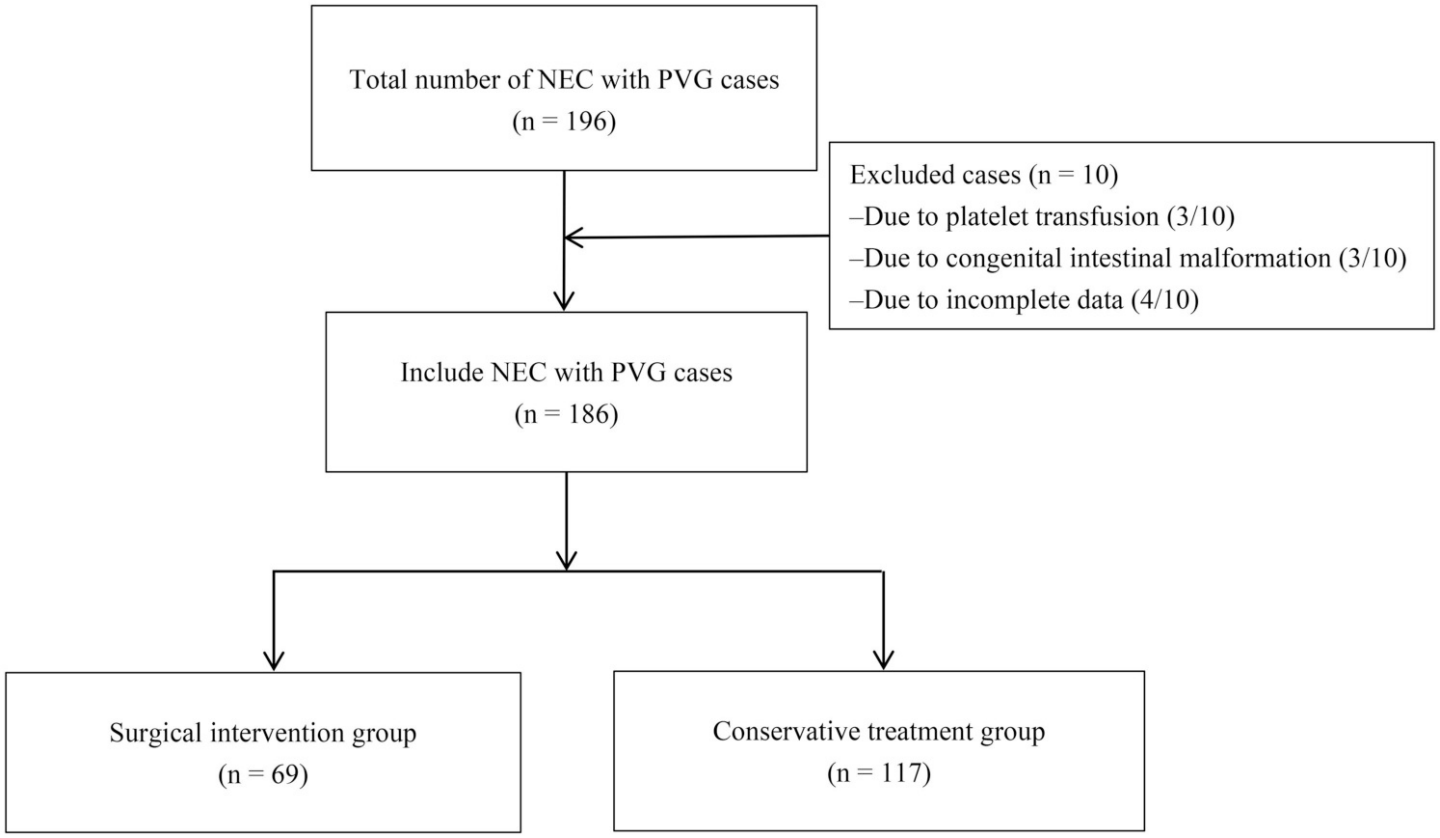
Patient enrollment flowchart for necrotizing enterocolitis neonates with portal venous gas.

A total of 186 NEC neonates with ultrasonographically confirmed PVG were included in this study. The patient enrollment process is summarized in Figure 1. The study cohort had the following characteristics at the time of PVG diagnosis: a mean postnatal age of 13.80 ± 9.70 days, a mean corrected gestational age of −4.27 ± 3.60 weeks, and a mean weight of 2,595.54 ± 782.40 g. Female predominance was observed in 54.30% (101/186) of patients. Assisted reproductive technology accounted for 16.13% (30/186) of conceptions, 70.97% (132/186) were delivered via cesarean section, and 29.57% (55/186) were from multiple gestations. Additional demographic characteristics and maternal perinatal factors are detailed in Table 1.

**Table 1:** Demographic and prenatal characteristics in necrotizing enterocolitis neonates with portal venous gas.

Variables	All (*n* = 186)	Surgical intervention (*n* = 69)	Conservative treatment (*n* = 117)	Statistics	*P*
Demographic characteristics					
Sex, *n* (%)				*χ*^2^ = 1.85	0.17
Male	85 (45.70)	36 (52.17)	49 (41.88)		
Female	101 (54.30)	33 (47.83)	68 (58.12)		
Mode of delivery, *n* (%)				*χ*^2^ = 1.82	0.18
Vaginal	54 (29.03)	16 (23.19)	38 (32.48)		
C-section	132 (70.97)	53 (76.81)	79 (67.52)		
Apgar 1 min, M (Q_1_, Q_3_)	9.00 (8.00, 10.00)	9.00 (8.00, 10.00)	9.00 (9.00, 10.00)	*Z* = −2.15	**0** **.** **03**
Apgar 5 min, M (Q_1_, Q_3_)	10.00 (10.00, 10.00)	10.00 (9.00, 10.00)	10.00 (10.00, 10.00)	*Z* = −3.48	**<0** **.** **01**
Apgar 10 min, M (Q_1_, Q_3_)	10.00 (10.00, 10.00)	10.00 (10.00, 10.00)	10.00 (10.00, 10.00)	*Z* = −2.08	**0** **.** **04**
Gestational age (weeks), M (Q_1_, Q_3_)	34.20 (31.43, 37.00)	31.57 (28.60, 33.40)	35.30 (33.60, 37.40)	*Z* = 6.66	**<0** **.** **01**
Corrected gestational age at PVG (weaks), mean ± SD	−4.27 ± 3.60	−6.37 ± 3.83	−3.03 ± 2.82	*t* = −6.83	**<0** **.** **01**
Weight at PVG (g), mean ± SD	2,595.54 ± 782.40	2,072.03 ± 702.93	2,904.27 ± 653.81	*t* = −8.15	**<0** **.** **01**
Maternal and prenatal characteristics					
Maternal age (years), M (Q_1_, Q_3_)	29.00 (26.00, 33.00)	30.00 (25.00, 34.00)	29.00 (26.00, 33.00)	*Z* = 0.04	0.97
Intrahepatic cholestasis, *n* (%)	10 (5.38)	4 (5.80)	6 (5.13)	*χ*^2^ = 0.00	1.00
Gestational diabetes mellitus, *n* (%)	27 (14.52)	9 (13.04)	18 (15.38)	*χ*^2^ = 0.19	0.66
Gestational hypertension, *n* (%)	14 (7.53)	7 (10.14)	7 (5.98)	*χ*^2^ = 1.08	0.30
Maternal hypothyroidism, *n* (%)	11 (5.91)	2 (2.90)	9 (7.69)	*χ*^2^ = 1.03	0.31
Multiple pregnancy, *n* (%)	55 (29.57)	32 (46.38)	23 (19.66)	*χ*^2^ = 14.88	**<0** **.** **01**
*In vitro* fertilization, *n* (%)	30 (16.13)	10 (14.49)	20 (17.09)	*χ*^2^ = 0.22	0.64
Meconium-stained amniotic fluid, *n* (%)	11 (5.91)	7 (10.14)	4 (3.42)	*χ*^2^ = 2.42	0.12
Premature rupture of membranes, *n* (%)	64 (34.41)	30 (43.48)	34 (29.06)	*χ*^2^ = 4.00	**0** **.** **05**
Antenatal corticosteroids, *n* (%)	70 (37.63)	32 (46.38)	38 (32.48)	*χ*^2^ = 3.57	0.06
Prenatal antibiotics exposure, *n* (%)	33 (17.74)	14 (20.29)	19 (16.24)	*χ*^2^ = 0.49	0.49

PVG, portal venous gas; *t*, *t*-test; *Z*, Mann–Whitney *U*-test; *χ*^2^, chi-square test; -, Fisher’s exact test; SD, standard deviation; M, median; Q_1_, 1st quartile; Q_3_, 3rd quartile.

Bold values indicate statistically significant differences (*P*<0.05) between groups.

Among the 186 neonates, the most common preoperative symptom was bloody stools, accounting for 75.27% (140/186). Concurrently, 84.41% (157/186) of the patients presented with diminished or absent bowel sounds. With respect to treatment modalities, 37.10% (69/186) of the patients underwent surgical intervention, with enterostomy performed in 92.75% (64/69) and peritoneal drainage in 7.25% (5/69) of surgical cases. The overall 30-day mortality was 13.98% (26/186). Additional clinical details are provided in Table 2.

**Table 2:** Comparison of clinical informations in necrotizing enterocolitis neonates with portal venous gas.

Variables	All (*n* = 186)	Surgical intervention (*n* = 69)	Conservative treatment (*n* = 117)	Statistics	*P*
Pre-NEC clinical information					
Atrial septal defect, *n* (%)	101 (54.30)	46 (66.67)	55 (47.01)	*χ*^2^ = 6.76	**0.01**
Ventricular septal defect, *n* (%)	12 (6.45)	6 (8.70)	6 (5.13)	*χ*^2^ = 0.42	0.52
Patent ductus arteriosus, *n* (%)	36 (19.35)	18 (26.09)	18 (15.38)	*χ*^2^ = 3.19	0.07
Bronchopulmonary dysplasia, *n* (%)	11 (5.91)	7 (10.14)	4 (3.42)	*χ*^2^ = 2.42	0.12
Red blood cell transfusion, *n* (%)	39 (20.97)	17 (24.64)	22 (18.80)	*χ*^2^ = 0.89	0.35
Exclusive breastfeeding, *n* (%)	37 (19.89)	7 (10.14)	30 (25.64)	*χ*^2^ = 6.54	**0** **.** **01**
Antibiotic use, *n* (%)	166 (89.25)	63 (91.30)	103 (88.03)	*χ*^2^ = 0.48	0.49
Clinical manifestations					
Vomiting, *n* (%)	77 (41.40)	27 (39.13)	50 (42.74)	*χ*^2^ = 0.23	0.63
Bloody stool, *n* (%)	140 (75.27)	49 (71.01)	91 (77.78)	*χ*^2^ = 1.07	0.30
Fever, *n* (%)	21 (11.29)	8 (11.59)	13 (11.11)	*χ*^2^ = 0.01	0.92
Diarrhea, *n* (%)	37 (19.89)	7 (10.14)	30 (25.64)	*χ*^2^ = 6.54	**0.01**
Abdominal distention, *n* (%)	76 (40.86)	22 (31.88)	54 (46.15)	*χ*^2^ = 3.66	0.06
Decreased bowel sounds, *n* (%)	157 (84.41)	61 (88.41)	96 (82.05)	*χ*^2^ = 1.33	0.25
Blood tests at the time of portal venous gas diagnosis					
White blood cell count (×10^9^/L), M (Q_1_, Q_3_)	10.07 (7.46, 13.30)	8.02 (4.91, 12.91)	10.76 (8.64, 13.38)	*Z* = −3.09	**<0.01**
Platelet count (×10^9^/L), M (Q_1_, Q_3_)	159.88 (108.64, 208.18)	116.48 (71.68, 165.76)	174.72 (127.12, 235.20)	*Z* = −5.46	**<0.01**
Hemoglobin level (g/dL), Mean ± SD	143.11 ± 32.49	127.17 ± 33.55	152.50 ± 28.00	*t* = −5.53	**<0.01**
Lymphocyte count (×10^9^/L), M (Q_1_, Q_3_)	3.79 (2.03, 5.29)	2.24 (1.37, 4.41)	4.37 (2.82, 5.63)	*Z* = −4.44	**<0.01**
Neutrophil count (×10^9^/L), M (Q_1_, Q_3_)	4.86 (3.07, 6.83)	4.04 (2.10, 7.56)	5.17 (3.49, 6.72)	*Z* = −2.14	**0.03**
C-reactive protein level (mg/L), M (Q_1_, Q_3_)	18.20 (14.56, 50.96)	40.04 (14.56, 78.26)	16.38 (12.74, 23.66)	*Z* = 5.81	**<0.01**
Platelet-to-lymphocyte ratio, M (Q_1_, Q_3_)	45.87 (29.54, 68.77)	47.62 (23.94, 80.37)	44.50 (30.78, 65.29)	*Z* = 0.09	0.93
Neutrophil-to-lymphocyte ratio, M (Q_1_, Q_3_)	1.31 (0.80, 2.65)	1.84 (0.73, 3.03)	1.11 (0.81, 2.10)	*Z* = 1.91	0.06
Lymphocyte-to-C-reactive protein ratio (×10^7^/mg), M (Q_1_, Q_3_)	14.94 (6.45, 30.77)	6.73 (2.75, 15.38)	23.12 (10.44, 35.71)	*Z* = −6.40	**<0.01**
Systemic immune-inflammation index (×10^9^/L), M (Q_1_, Q_3_)	187.30 (106.89, 365.33)	157.12 (72.19, 378.23)	214.05 (139.10, 358.30)	*Z* = −1.89	0.06
Platelet-to-C-reactive protein ratio (×10^9^/mg), M (Q_1_, Q_3_)	6.36 (3.43, 11.71)	2.91 (1.64, 4.61)	8.57 (5.81, 13.43)	*Z* = −7.82	**<0.01**
Clinical outcomes					
30-day mortality, *n* (%)	26 (13.98)	21 (30.43)	5 (4.27)	*χ*^2^ = 24.70	**<0.01**

*t*, *t*-test; *Z*, Mann–Whitney *U*-test; *χ*^2^, chi-square test; -, Fisher’s exact test; SD, standard deviation; M, median; Q_1_, 1st quartile; Q_3_, 3rd quartile.

Bold values indicate statistically significant differences (*P*<0.05) between groups.

### Group characteristics based on treatment methods for NEC patients with PVG

Patients were divided into two groups: the surgical intervention group (*n* = 69) and the conservative treatment group (*n* = 117). Compared to the conservative group, the surgical group had lower 5-min Apgar scores, gestational age at PVG diagnosis, weight at PVG diagnosis, and exclusive breastfeeding rates (all *P*'s < 0.05) but higher rates of multiple gestation (*P* < 0.01), premature rupture of membranes (*P* = 0.05), and atrial septal defects (*P* = 0.01). Hematologically, the surgical group showed lower leukocyte, platelet, hemoglobin, absolute lymphocyte, and absolute neutrophil counts (all *P*'s < 0.05) but higher CRP levels (*P* < 0.01). Regarding composite markers, both the lymphocyte-to-C-reactive protein ratio (LCR) and PCR were also lower in the surgical group (both *P*'s < 0.01), whereas platelet-to-lymphocyte ratio, neutrophil-to-lymphocyte ratio, and systemic immune-inflammation index showed no significant intergroup differences (all *P*'s > 0.05) (Tables 1, 2).

### Predictive value of hematological parameters for surgical intervention in NEC patients with PVG

To evaluate the independent associations between hematologic parameters and surgical intervention risk in NEC patients with PVG, we performed multivariable logistic regression. The model was adjusted for 5-min Apgar score, corrected gestational age and weight at PVG diagnosis, multiple gestation, premature rupture of membranes, exclusive breastfeeding, and atrial septal defect. The regression models satisfied linearity assumptions and demonstrated adequate goodness of fit (Hosmer–Lemeshow test, *P* = 0.17; Supplementary Figure S1), with all VIF values below 2.41 (Supplementary Table S1), indicating no substantial multicollinearity. The models were internally validated with 2,000 bootstrap resamples, showing excellent discriminative ability (AUC 0.89; bootstrap-corrected AUC 0.87; Supplementary Table S3). After adjustment, the platelet count, hemoglobin level, absolute lymphocyte count, CRP, LCR, and PCR remained independently associated with the risk of surgical intervention (Table 3).

**Table 3:** Adjusted odds ratios for surgical intervention in necrotizing enterocolitis with portal venous gas: multivariable logistic regression.

Variables	*β*	S.E.	*Z*	*P*	OR (95% CI)
White blood cell count (×10^9^/L)	−0.04	0.03	−1.38	0.19	0.96 (0.90–1.02)
Platelet count (×10^9^/L)	−0.01	0.00	−3.10	**<0.01**	0.99 (0.99–0.99)
Hemoglobin level (g/dL)	−0.03	0.01	−3.82	**<0.01**	0.97 (0.96–0.99)
Lymphocyte count (×10^9^/L)	−0.18	0.08	−2.19	**0.03**	0.84 (0.72–0.98)
Neutrophil count (×10^9^/L)	−0.02	0.04	−0.56	0.573	0.98 (0.90–1.06)
C-reactive protein level (mg/L)	0.04	0.01	4.24	**<0.01**	1.04 (1.02–1.06)
Lymphocyte-to-C-reactive protein ratio (×10^7^/mg)	−0.05	0.01	−3.15	**<0.01**	0.95 (0.93–0.98)
Platelet-to-C-reactive protein ratio (×10^9^/mg)	−0.26	0.06	−4.55	**<0.01**	0.77 (0.69–0.86)

Adjusted for weight at PVG (g), corrected gestational age at PVG (w), Apgar score (5 min), platelet-to-C-reactive protein ratio (×10^9^/mg), exclusive breastfeeding, premature rupture of membranes, multiple pregnancy, and atrial septal defect. OR, odds ratio; CI, confidence interval.

Bold values indicate statistically significant differences (*P*<0.05) between groups.

To further assess the predictive capacity of hematologic parameters, we performed ROC curve analysis with comparisons using DeLong's test. The PCR achieved the highest predictive performance for surgical intervention (AUC 0.84, 95% CI: 0.78–0.91). This result significantly outperformed individual parameters including lymphocyte count, hemoglobin, platelet count, and C-reactive protein alone (all *P*'s < 0.05). Although the LCR also showed good discriminative ability (AUC 0.78), the superior predictive capacity of the PCR approached statistical significance (*P* = 0.08), establishing it as the most robust routine biomarker in this cohort (Table 4).

**Table 4:** Comparison of predictive performance by DeLong's test for surgical intervention in necrotizing enterocolitis with portal venous gas.

Predictor	AUC (95% CI)	Sensitivity (95% CI)	Specificity (95% CI)	Cut off	*P*
Hematological markers					
Lymphocyte count (×10^9^/L)	0.70 (0.61–0.78)	0.71 (0.60–0.82)	0.67 (0.58–0.75)	3.44	**<0.01**
Hemoglobin level (g/dL)	0.73 (0.65–0.80)	0.51 (0.39–0.63)	0.90 (0.84–0.95)	117.50	**0.01**
Platelet count (×10^9^/L)	0.74 (0.66–0.82)	0.70 (0.59–0.80)	0.67 (0.60–0.77)	152.60	**0.01**
C-reactive protein level (mg/L)	0.75 (0.68–0.83)	0.65 (0.54–0.76)	0.74 (0.66–0.82)	20.93	**<0.01**
Lymphocyte-to-C-reactive protein ratio (×10^7^/mg)	0.78 (0.71–0.85)	0.67 (0.56–0.78)	0.79 (0.72–0.87)	9.48	0.08
Platelet-to-C-reactive protein ratio (×10^9^/mg)	0.84 (0.78–0.91)	0.80 (0.70–0.89)	0.85 (0.79–0.92)	4.84	Reference

AUC, area under the receiver operating characteristic curve. *P* values were derived from DeLong's test for comparison of the AUC of each predictor against the reference predictor.

Bold values indicate statistically significant differences (*P*<0.05) between groups.

The ROC curve analysis also identified an optimal serum PCR cutoff of ≤4.84 × 10^9^/mg for predicting surgical intervention, with a sensitivity of 80.0% (95% CI: 70%–89.0%) and a specificity of 85.0% (95% CI: 79.0%–92.0%; Table 4). To evaluate the robustness of this threshold and its performance across Bell's stages, we conducted sensitivity analyses across subgroups stratified by gestational age, weight at PVG, and Bell's stage (stage IIb). The PCR maintained consistently strong performance across all subgroups (AUC range: 0.76–0.86), with subgroup-specific optimal cutoff values (range: 4.41–4.92 × 10^9^/mg) closely aligned with the original value, demonstrating its stability and generalizability (Table 5).

**Table 5:** Sensitivity analysis of the PCR for surgical intervention across different subgroups.

Group	*n*	AUC	Optimal threshold	Sensitivity	Specificity	Youden index
By gestational age						
Low GA (23.40–32.40 weeks)	62.00	0.86	4.84	0.86	0.90	0.76
Medium GA (32.40–36.00 weeks)	62.00	0.76	4.91	0.67	0.91	0.58
High GA (36.00–39.60 weeks)	62.00	0.81	4.42	0.78	0.96	0.74
By weight at PVG						
Low weight (760.00–2,170.00 g)	63.00	0.82	4.84	0.80	0.89	0.69
Medium weight (2,170.00–2,879.00 g)	61.00	0.84	4.92	0.81	0.89	0.70
High weight (2,879.00–4,880.00 g)	62.00	0.84	4.41	0.75	0.98	0.73
Overall	186.00	0.84	4.84	0.80	0.85	0.65

**Figure 2:**
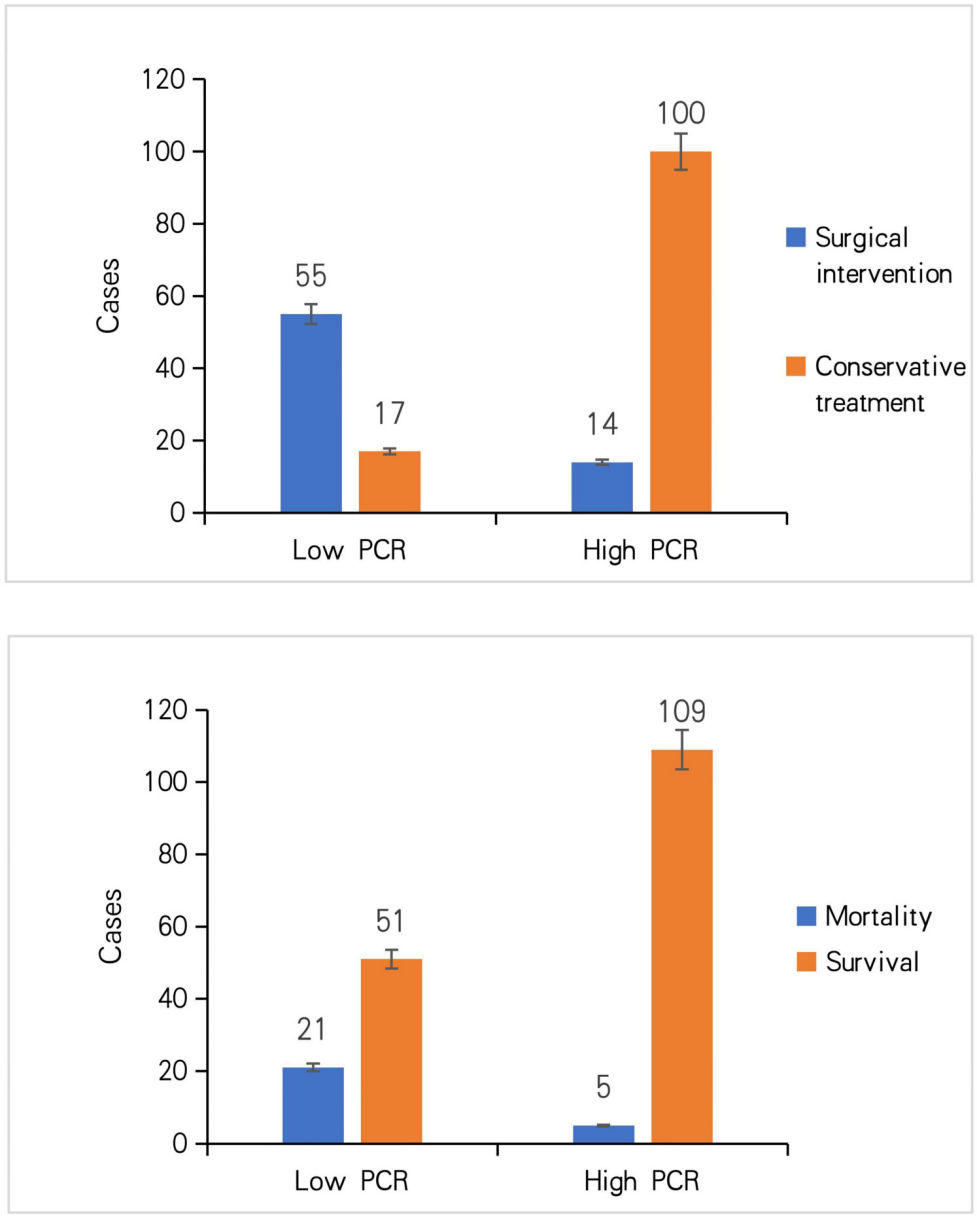
Comparative analysis of surgical and mortality outcomes between low- (≤4.84 × 10^9^/mg) and high-PCR (>4.84 × 10^9^/mg) groups.

Using the optimal PCR cutoff of 4.84 × 10^9^/mg, patients were stratified into low-PCR (≤4.84 × 10^9^/mg) and high-PCR (>4.84 × 10^9^/mg) groups. The surgical intervention rate was significantly higher in the low-PCR group (76.39% vs. 12.28%, *P* < 0.01). Moreover, the mortality rate was also significantly higher in the low-PCR group (29.17% vs. 4.39%, *P* < 0.01), underscoring the clinical significance of the PCR for risk stratification beyond surgical intervention alone (Figure 2).

### Predictive value of the PCR for mortality in NEC patients with PVG

**Figure 3:**
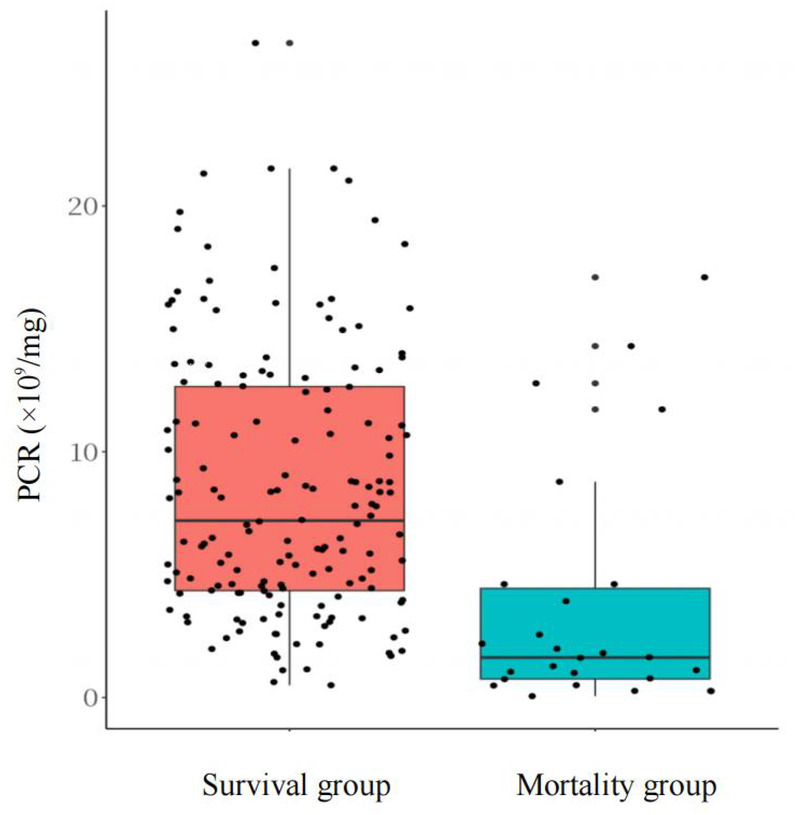
Distribution of PCR values between survival and mortality groups.

To further evaluate the potential predictive value of the PCR for mortality in NEC patients with PVG, we first conducted a systematic cohort analysis comparing deceased and surviving patients. Univariate analysis showed significantly lower PCR values in the mortality group (Figure 3), with additional intergroup differences detailed in Supplementary Table S7. We then constructed a multivariable logistic regression model adjusted for key clinical confounders. The model satisfied linearity assumptions, demonstrated adequate goodness of fit, and was internally validated with 2,000 bootstrap resamples (Supplementary Tables 2, 4; Supplementary Figure S2). After adjustment, the PCR maintained an independent inverse association with mortality risk (adjusted OR = 0.80, 95% CI: 0.69–0.92, *P* < 0.01; Table 6).

**Table 6:** Adjusted odds ratios of the PCR for mortality in necrotizing enterocolitis with portal venous gas: multivariable logistic regression.

Variables	*β*	S.E	*Z*	*P*	OR (95% CI)
Platelet-to-C-reactive protein ratio (×10^9^/mg)	−0.23	0.08	−3.01	**<0.01**	0.80 (0.69–0.92)

Adjusted for gestational age (w), platelet-to-C-reactive protein ratio (×109/mg), premature rupture of membranes, multiple pregnancy, antenatal corticosteroids, and *in vitro* fertilization. OR, odds ratio; CI, confidence interval.

Bold values indicate statistically significant differences (*P*<0.05) between groups.

ROC curve analysis further supported the predictive capacity of the PCR for mortality, yielding an AUC of 0.80 (95% CI: 0.69–0.92). At the optimal cutoff value of ≤2.57 × 10^9^/mg, the PCR was associated with mortality with a sensitivity of 69.0% (95% CI: 51.0%–87.0%) and a specificity of 91.0% (95% CI: 87.0%–96.0%) (Table 7). Sensitivity analysis revealed that the PCR maintained good to excellent predictive performance for mortality across most subgroups (Table 8). After stratification by gestational age (GA), the PCR demonstrated excellent discriminative ability in the most-preterm neonates (AUC = 0.85), with an optimal cutoff of 2.57 × 10^9^/mg providing balanced sensitivity (80%) and specificity (83%). In the moderate-gestational-age group (AUC = 0.76), the optimal cutoff was 3.67 × 10^9^/mg, while the low-birth-weight subgroup (AUC = 0.80) showed comparably strong predictive performance using the same cutoff of 2.57 × 10^9^/mg as the most-preterm group. Furthermore, the PCR maintained a reliable predictive value in the predominant Bell's stage IIb subgroup (AUC = 0.81), with an optimal cutoff (2.62 × 10^9^/mg) nearly identical to the overall value. Importantly, the overall optimal cutoff of 2.57 × 10^9^/mg remained consistent across several key high-risk subgroups, including the most-preterm and low-birth-weight groups, suggesting its potential stability as an objective auxiliary indicator for mortality risk stratification in this vulnerable population.

**Table 7:** ROC analysis of the PCR for predicting mortality in necrotizing enterocolitis with portal venous gas.

Predictor	AUC (95% CI)	Sensitivity (95% CI)	Specificity (95% CI)	Cut off
Platelet-to-C-reactive protein ratio (×10^9^/mg)	0.80 (0.69–0.92)	0.69 (0.51–0.87)	0.91 (0.87–0.96)	2.57

AUC, area under the receiver operating characteristic curve.

**Table 8:** Sensitivity analysis of the PCR for mortality across different subgroups.

Group	*n*	AUC	Optimal threshold	Sensitivity	Specificity	Youden index
By gestational age						
Low GA (23.40–32.40 weeks)	62.00	0.85	2.57	0.80	0.83	0.63
Medium GA (32.40–36.00 weeks)	62.00	0.76	3.67	0.80	0.81	0.61
High GA (36.00–39.60 weeks)	62.00	0.67	2.96	0.50	0.95	0.45
By weight at PVG						
Low weight (760.00–2,170.00 g)	63.00	0.80	2.57	0.79	0.84	0.62
Medium weight (2,170.00–2,879.00 g)	61.00	0.89	3.92	1.00	0.77	0.79
High weight (2,879.00–4,880.00 g)	62.00	0.76	2.42	0.57	0.98	0.55
By Bell's stage						
Bell's stage IIb	178.00	0.81	2.62	0.67	0.93	0.60
Overall	186.00	0.80	2.57	0.69	0.91	0.61

## Discussion

Neonatal PVG, as one of the most severe complications of necrotizing enterocolitis (NEC), has traditionally been regarded as a marker of intestinal necrosis and disease progression, often considered a crucial indication for surgical intervention. However, accumulating clinical evidence reveals significant individual variability in the clinical manifestations of PVG patients. Our study found that approximately 37.10% (69/186) of NEC neonates with PVG ultimately required surgical treatment, with an overall mortality rate of 13.98% (26/186), which is largely consistent with the findings reported by Chen et al. (surgical rate 41.8%, mortality 15.7%) (7). These results suggest that PVG should not be considered an absolute surgical indication for NEC, and support a re-evaluation of its role in NEC diagnosis and treatment. We hypothesize that advancements in neonatal intensive care technologies and imaging diagnostics may evolve the clinical significance of PVG. Consequently, the determination of surgical indications for NEC neonates with PVG may benefit from additional objective clinical indicators.

Based on the characteristic pathophysiological mechanisms of NEC, we focused on two critical pathological processes: coagulation dysfunction (reflected by platelet activation/consumption) and systemic inflammatory response (indicated by elevated CRP levels). Existing studies have conclusively demonstrated that intestinal ischemic injury during NEC progression leads to platelet activation, adhesion, and abnormal microcirculatory aggregation, subsequently triggering disseminated intravascular coagulation (DIC) and ultimately causing thrombocytopenia (15–17). Multiple clinical studies have confirmed that platelet reduction is significantly associated with the severity of intestinal damage and poor prognosis in NEC (17–19). Concurrently, research evidence indicates that intestinal mucosal injury and bacterial translocation can activate systemic inflammatory cascades, promoting CRP gene transcription in hepatocytes through signaling pathways such as NF-κB (20, 21). As a crucial acute-phase inflammatory protein, variations in CRP levels have been widely used for monitoring NEC progression (22, 23). However, both isolated platelet count and CRP measurements demonstrate significant limitations in clinical predictive value. This understanding led us to propose an innovative hypothesis: the PCR, integrating information on both coagulation and inflammatory responses, may provide more accurate predictive value for clinical assessment of NEC.

Our study systematically evaluated the clinical utility of the PCR in NEC patients with PVG through two key clinical outcomes: surgical intervention and 30-day mortality. In predicting the need for surgical intervention, multivariate analysis demonstrated that the PCR maintained an independent association with the need for surgical intervention after adjusting for potential confounders, with a 23% reduction in surgical risk per unit increment (adjusted odds ratio (aOR) = 0.77, 95% CI: 0.69–0.86, *P* < 0.01). ROC analysis revealed higher predictive performance of the PCR (AUC = 0.84, 95% CI: 0.78–0.91) compared to the isolated platelet count (AUC = 0.74), CRP level (AUC = 0.75), or other combined parameters like the LCR (AUC = 0.78). At the optimal cutoff value of ≤4.84 × 10^9^/mg, the PCR showed a sensitivity of 80% and specificity of 85% for predicting surgical indications. For 30-day mortality prediction, the PCR demonstrated to have good prognostic value (AUC = 0.80, 95% CI: 0.69–0.92), with 69% sensitivity and 91% specificity at an optimal cutoff value of ≤2.57 × 10^9^/mg. These results lend support to the clinical value of the PCR as a composite biomarker, outperforming individual parameters in stratifying risk for both surgical decision-making and prognostic assessment. These findings are pathophysiologically consistent with the characteristic mechanisms of NEC' involve microcirculatory dysfunction and systemic inflammatory cascades.

An important consideration is the heterogeneity within the NEC population, particularly regarding variations in body weight and gestational age, which raises the question of whether the PCR maintains consistent predictive performance across these subgroups. Our sensitivity analyses provided reassuring evidence in this regard. When the cohort was stratified into tertiles based on weight and gestational age to ensure statistically balanced subgroups, the PCR demonstrated robust predictive performance with a stable cutoff value for surgical intervention across all strata. For mortality prediction, while greater variability in performance was observed across some subgroups, the original optimal cutoff value (≤2.57 × 10^9^/mg) remained consistent with those identified in key high-risk subgroups—specifically the lowest gestational age (23.40–32.40 weeks) and lowest weight (760.00–2,170.00 g)—supporting its robustness and clinical utility, particularly in vulnerable neonatal populations. The variability observed in other subgroups may be attributed to the limited sample size inherent in this retrospective study and warrants further investigation in larger cohorts.

Accumulating evidence from recent studies has further validated the clinical application of the PCR as a prognostic biomarker. In neonatal sepsis, Engade et al. (*n* = 120) reported significantly lower PCR values in septic vs. non-septic infants on day 3 (1.49 vs. 4.5 × 10^9^/mg) and day 5 (0.97 vs. 4.3 × 10^9^/mg) (*P* = 0.04) (24); Jin et al. (*n* = 1,385) further identified that the PCR serves as an independent predictor of sepsis, exhibiting better predictive performance (AUC 0.73) compared to CRP (AUC 0.68) or platelet count alone (AUC 0.67) (12). In COVID-19, Djordjevic et al. (*n* = 612) observed lower PCR values in severe fatal cases than in mild cases (2.0 vs. 2.7 × 10^9^/mg; *P* < 0.01) (25), while Qiao et al. (*n* = 76) demonstrated its predictive utility for severe disease (AUC 0.84) and multiorgan injury (AUC 0.72) (14). In chronic heart failure, Powrózek et al. (*n* = 154) reported that the PCR is a significant predictor of disease severity and survival (13). Similarly, in hematopoietic stem cell transplantation, Nakajima et al. (*n* = 152) found that low pretransplant PCR values independently predicted poorer overall survival (HR = 2.77, *P* = 0.01) and higher non-relapse mortality (aOR = 2.79, *P* = 0.02) (26). These findings corroborate our observations in NEC patients with PVG, collectively demonstrating the predictive value of the PCR.

Furthermore, compared to other biomarkers such as the C-reactive protein/albumin ratio, lactate, intestinal fatty acid-binding protein, and fecal calprotectin, the PCR offers advantages including simpler detection, lower cost, higher reproducibility, and greater stability. These characteristics make it more suitable for widespread adoption across healthcare institutions at all levels, facilitating early risk stratification in NEC patients with PVG. Therefore, based on our study findings, we propose that the PCR could be considered a routine monitoring parameter for NEC infants with PVG, using 4.84 × 10^9^/mg as a potential critical threshold for risk stratification. For high-risk infants with PCR values below this cutoff value, we suggest intensifying clinical monitoring frequency and proactively assembling a multidisciplinary team to evaluate surgical indications. This PCR-based objective assessment model could provide auxiliary quantitative evidence for clinical decision-making and merits investigation in future studies to determine if it can improve outcomes for these high-risk infants.

To our knowledge, our study provides several new contributions to the field: (1) the first systematic evaluation of the PCR as a composite inflammatory marker in patients with NEC and PVG; (2) identification of a potentially clinically useful predictive threshold (4.84 × 10^9^/mg); and (3) preliminary exploration of a novel dynamic PCR monitoring approach for NEC-PVG management. These findings may offer new insights into the coagulation–inflammation axis in NEC while proposing a quantifiable parameter that could potentially serve as an auxiliary tool in clinical decision-making; however, prospective validation is required before clinical implementation.

### Limitations

This study has several limitations that should be acknowledged. First, as a single-center retrospective study, it may be subject to selection bias, and the sample size remains limited. Second, PVG is a dynamic and potentially transient finding. Due to the retrospective nature of our study, we were unable to precisely ascertain the time interval between the clinical onset of NEC (meeting Bell's stage ≥ IIa criteria) and the first ultrasonographic detection of PVG for all patients. This lack of data, coupled with the elusive nature of PVG, may potentially influence the representativeness of our study cohort. Third, although we adjusted for key confounders and conducted sensitivity analyses, the potential for residual confounding inherent to observational designs persists. Fourth, as a referral center study, detailed therapeutic data—particularly the total duration of antibiotic therapy prior to enrollment—were unavailable for a subset of transferred patients. Consequently, “antibiotic use” was used as a surrogate measure, which may not fully capture the impact of treatment duration. In addition, our analysis was based solely on PCR values at diagnosis and did not assess the prognostic value of dynamic PCR changes. Most importantly, the proposed cutoff value of 4.84 × 10^9^/mg requires validation through multicenter, large-scale prospective studies.

Future research should focus on several key directions: validating the predictive performance of the PCR in multicenter prospective cohorts; evaluating the utility of serial PCR measurements in tracking disease progression; and systematically assessing the integration of the PCR with emerging biomarkers to optimize predictive accuracy; and establishing prospective studies with standardized imaging protocols to delineate the precise timing of PVG onset relative to NEC progression and its prognostic significance.

### Summary

This study systematically analyzed clinical data from 186 NEC neonates with PVG, and for the first time evaluated that the PCR may serve as a promising indicator for predicting both the need for surgical intervention and prognosis. The results demonstrated that a PCR value ≤ 4.84 × 10^9^/mg was significantly associated with surgical intervention requirement (AUC = 0.84) and with 30-day mortality (AUC = 0.80). As a composite biomarker integrating information about both coagulation dysfunction and systemic inflammatory response, the PCR showed superior predictive performance compared to single parameters, while being simple and cost-effective. Based on these findings, we suggest that PCR testing could be incorporated into routine monitoring for NEC-PVG patients, using 4.84 × 10^9^/mg as a potential risk stratification threshold, and propose enhanced monitoring and timely surgical evaluation for patients with low PCR values. Our study identified a clinically applicable predictive threshold and proposed a novel dynamic monitoring-based management approach. These findings provide supporting evidence to inform clinical decision-making and warrant further investigation to assess their potential to improve the diagnosis, treatment, and outcomes of this high-risk population. Prospective multicenter validation is required before clinical implementation.

## Data Availability

The original contributions presented in the study are included in the article/Supplementary Material, further inquiries can be directed to the corresponding author.
