# Supplementary material for: Platelet-to-C-reactive protein ratio stratifies surgical risk and mortality in necrotizing enterocolitis neonates with portal venous gas
Source: Front Pediatr. 2026 Jan 2;13:1686076. doi: 10.3389/fped.2025.1686076 (PMC12808459; doi:10.3389/fped.2025.1686076)
Supplement: Supplementary file 1 [file Supplementaryfile1.docx]

**
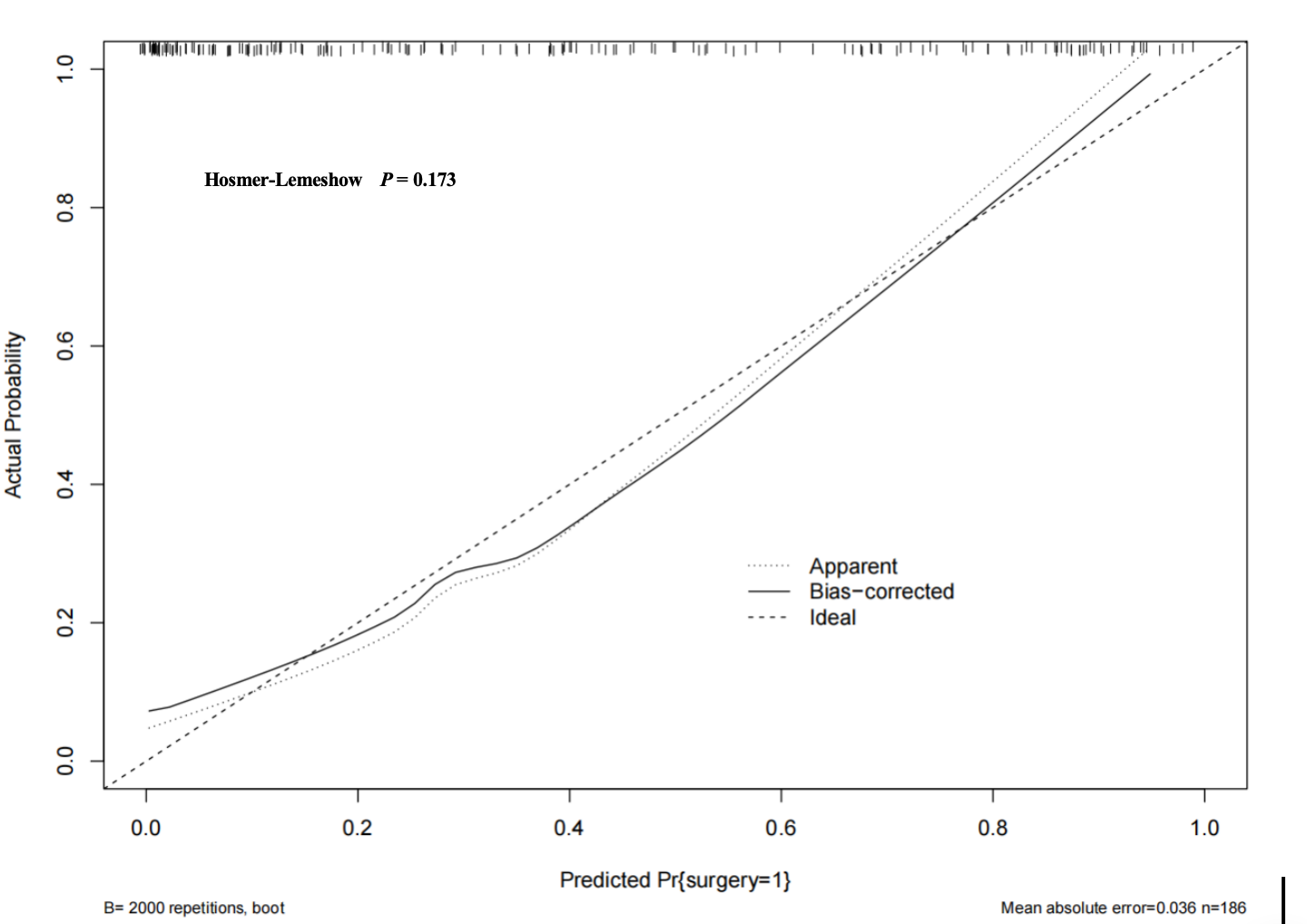
**

**Supplementary Figure 1. Calibration curve of the model for predicting Surgical Intervention assessed by the Hosmer-Lemeshow test with bootstrap validation**


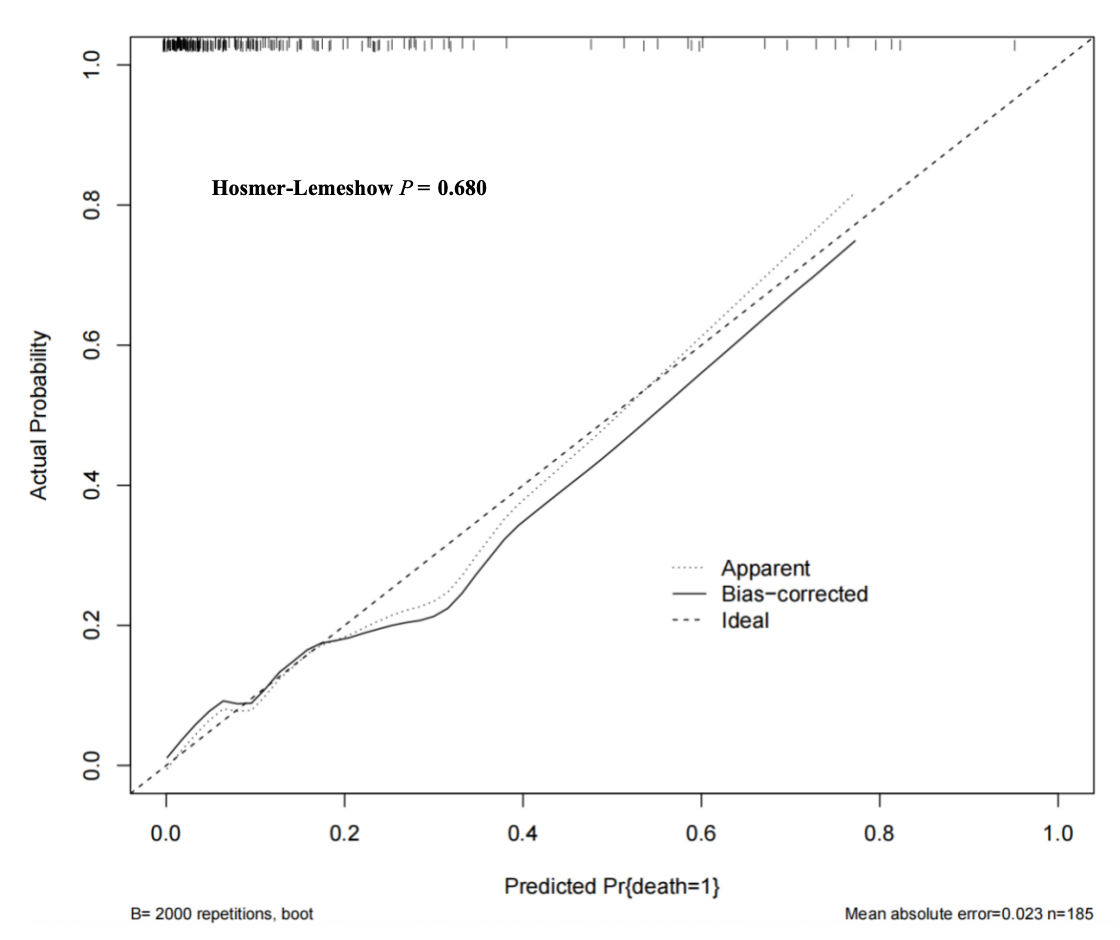


**Supplementary Figure 2. Calibration curve of the model for predicting Mortality assessed by the Hosmer-Lemeshow test with bootstrap validation**
