# Supplementary material for: Platelet-to-C-reactive protein ratio stratifies surgical risk and mortality in necrotizing enterocolitis neonates with portal venous gas
Source: Front Pediatr. 2026 Jan 2;13:1686076. doi: 10.3389/fped.2025.1686076 (PMC12808459; doi:10.3389/fped.2025.1686076)
Supplement: Supplementary file 2 [file Supplementaryfile2.docx]

**Supplementary Table 1. Predictors of Surgical Intervention in Necrotizing Enterocolitis with Portal Venous Gas: Multivariable Logistic Regression with Multicollinearity Assessment**

| Variables | β | S.E | Z | *P* | OR (95% CI) | VIF |
| --- | --- | --- | --- | --- | --- | --- |
| Intercept | 4.68 | 2.44 | 1.92 | 0.05 | NA | NA |
| Weight at PVG (g) | -0.01 | 0.00 | -2.32 | **0.02** | 0.99 (0.98 - 1.00) | 2.41 |
| Corrected gestational age at PVG (w) | -0.05 | 0.09 | -0.48 | 0.63 | 0.95 (0.79 - 1.15) | 2.26 |
| Apgar score (5-min) | -0.14 | 0.18 | -0.78 | 0.44 | 0.87 (0.62 - 1.23) | 1.06 |
| Platelet-to-C-reactive protein ratio (×10^9^/mg) | -0.26 | 0.06 | -4.55 | **< 0.01** | 0.77 (0.69 - 0.86) | 1.05 |
| Exclusive breastfeeding | -0.94 | 0.56 | -1.67 | 0.10 | 0.39 (0.13 - 1.18) | 1.05 |
| Premature rupture of membranes | 0.85 | 0.44 | 1.95 | **0.05** | 2.34 (1.00 - 5.50) | 1.04 |
| Multiple pregnancy | 0.02 | 0.47 | 0.04 | 0.97 | 1.02 (0.41 - 2.54) | 1.16 |
| Atrial septal defect | 0.67 | 0.44 | 1.54 | 0.13 | 1.96 (0.83 - 4.62) | 1.10 |
| Abbreviations: CI, confidence interval; OR, odds ratio; S.E., standard error; VIF, variance inflation factor. | | | | | | |
| Note: All variables with VIF < 5 were retained, indicating no substantial multicollinearity. | | | | | | |

**Supplementary Table 2. Predictors of Mortality in Necrotizing Enterocolitis with Portal Venous Gas: Multivariable Logistic Regression with Multicollinearity Assessment**

| Variables | β | S.E | Z | *P* | OR (95% CI) | VIF |
| --- | --- | --- | --- | --- | --- | --- |
| Intercept | 1.70 | 2.25 | 0.74 | 0.45 | NA | NA |
| Gestational age (w) | -0.13 | 0.07 | -1.84 | **0.07** | 0.88 (0.77 - 1.01) | 1.22 |
| Platelet-to-C-reactive protein ratio (×10^9^/mg) | -0.19 | 0.07 | -2.71 | **0.01** | 0.82 (0.72 - 0.95) | 1.24 |
| Premature rupture of membranes | 1.70 | 0.56 | 3.06 | **< 0.01** | 5.46 (1.84 - 16.20) | 1.08 |
| Multiple pregnancy | 0.13 | 0.54 | 0.23 | 0.82 | 1.14 (0.39 - 3.30) | 1.22 |
| Antenatal corticosteroids | 1.29 | 0.56 | 2.31 | **0.02** | 3.65 (1.22 - 10.93) | 1.13 |
| In vitro fertilization | 1.83 | 0.63 | 2.90 | **< 0.01** | 6.23 (1.81 - 21.50) | 1.02 |
| Abbreviations: CI, confidence interval; OR, odds ratio; S.E., standard error; VIF, variance inflation factor. | | | | | | |
| Note: All variables with VIF < 5 were retained, indicating no substantial multicollinearity. | | | | | | |

**Supplementary Table 3. Predictive Performance of the Model for Surgical Intervention with Bootstrap Validation**

| AUC | Bootstrap-Corrected AUC | Bootstrap 95% CI | Accuracy (95% CI) | Sensitivity (95% CI) | Specificity (95% CI) | Cut-off |
| --- | --- | --- | --- | --- | --- | --- |
| 0.89 | 0.87 | 0.84 - 0.94 | 0.83 (0.77 - 0.88) | 0.84 (0.75 - 0.93) | 0.82 (0.75 - 0.89) | 147.93 |
| Bootstrap results are based on 2000 replicates. AUC = area under the receiver operating characteristic curve; CI = confidence interval. | | | | | | |

**Supplementary Table 4. Predictive Performance of the Model for Mortality with Bootstrap Validation**

| AUC | Bootstrap-Corrected AUC | Bootstrap 95% CI | Accuracy (95% CI) | Sensitivity (95% CI) | Specificity (95% CI) | Cut-off |
| --- | --- | --- | --- | --- | --- | --- |
| 0.86 | 0.81 | 0.78 - 0.93 | 0.82 (0.75 - 0.87) | 0.77 (0.61 - 0.93) | 0.83 (0.76 - 0.88) | 133.10 |
| Bootstrap results are based on 2000 replicates. AUC = area under the receiver operating characteristic curve; CI = confidence interval. | | | | | | |

**Supplementary Table 5. Adjusted Odds Ratio of PCR for Surgical Intervention at Bell’s Stage IIb**

| Variables | β | S.E | Z | *P* | OR (95%CI) |
| --- | --- | --- | --- | --- | --- |
|  |  |  |  |  |  |
| Platelet-to-C-reactive protein ratio (×10^9^/mg) | -0.23 | 0.05 | -4.14 | **<0.01** | 0.80 (0.72 - 0.88) |
| Adjust: Weight at PVG (g), Corrected gestational age at PVG (w), Apgar score (5-min), Exclusive Breastfeeding, Premature rupture of membranes, Multiple pregnancy, Atrial septal defect | | | | | |
| OR: Odds Ratio, CI: Confidence Interval | | | | | |

**Supplementary Table 6. Adjusted Odds Ratio of PCR for Mortality at Bell’s Stage IIb**

| Variables | β | S.E | Z | *P* | OR (95%CI) |
| --- | --- | --- | --- | --- | --- |
|  |  |  |  |  |  |
| Platelet-to-C-reactive protein ratio (×10^9^/mg) | -0.25 | 0.06 | -4.29 | **<0.01** | 0.78 (0.70-0.87) |
| Adjust: Gestational age (w), Premature rupture of membranes, Multiple pregnancy, Antenatal corticosteroids, In vitro fertilization | | | | | |
| OR: Odds Ratio, CI: Confidence Interval | | | | | |

**Supplementary Table 7. Comparison of Demographic, Prenatal, and Clinical Characteristics between Survival and Mortality Groups**

| Variables | All  (n = 186) | Survival  (n = 160) | Mortality  (n = 26) | Statistic | *P* | |  |
| --- | --- | --- | --- | --- | --- | --- | --- |
| **Demographic Characteristics** | | | | | | |  |
| Sex, n(%) |  |  |  | χ² = 0.81 | 0.37 | |  |
| Male | 85 (45.70) | 71 (44.38) | 14 (53.85) |  |  | |  |
| Female | 101 (54.30) | 89 (55.62) | 12 (46.15) |  |  | |  |
| Mode of delivery, n (%) |  |  |  | χ² = 2.59 | 0.11 | |  |
| Vaginal | 54 (29.03) | 43 (26.88) | 11 (42.31) |  |  | |  |
| C-section | 132 (70.97) | 117 (73.12) | 15 (57.69) |  |  | |  |
| Apgar 1min, M(Q_1_,Q_3_) | 9.00 (8.00, 10.00) | 9.00 (8.00, 10.00) | 9.00 (8.00, 9.00) | Z = 1.80 | 0.07 | |  |
| Apgar 5min, M(Q_1_,Q_3_) | 10.00 (10.00, 10.00) | 10.00 (10.00, 10.00) | 10.00 (9.00, 10.00) | Z = 1.84 | 0.07 | |  |
| Apgar 10min, M(Q_1_,Q_3_) | 10.00 (10.00, 10.00) | 10.00 (10.00, 10.00) | 10.00 (10.00, 10.00) | Z = 1.02 | 0.31 | |  |
| Gestational age (w), M(Q_1_,Q_3_) | 34.20 (31.43, 37.00) | 34.50 (32.12, 37.00) | 30.00 (26.93, 33.63) | Z = -2.68 | | **< 0.01** | |
| Corrected gestational age at PVG (w)  , Mean ± SD | -4.27 ± 3.60 | -3.98 ± 3.31 | -6.07 ± 4.77 | t = 2.15 | **0.04** | |  |
| Weight at PVG (g), Mean ± SD | 2595.54 ± 782.40 | 2642.88 ± 758.29 | 2304.23 ± 877.66 | t = 2.06 | **0.04** | |  |
| **Maternal and Prenatal Characteristics** | | | | | | |  |
| Maternal age(w), M(Q_1_,Q_3_) | 29.00 (26.00, 33.00) | 29.00 (26.00, 33.00) | 30.00 (26.50, 34.00) | Z = -0.80 | 0.42 | |  |
| Intrahepatic cholestasis, n(%) | 10 (5.38) | 10 (6.25) | 0 (0.00) | χ² = 0.71 | 0.40 | |  |
| Gestational diabetes mellitus, n(%) | 27 (14.52) | 22 (13.75) | 5 (19.23) | χ² = 0.19 | 0.66 | |  |
| Gestational hypertension, n(%) | 14 (7.53) | 13 (8.12) | 1 (3.85) | χ² = 0.13 | 0.71 | |  |
| Maternal Hypothyroidism, n(%) | 11 (5.91) | 11 (6.88) | 0 (0.00) | χ² = 0.87 | 0.35 | |  |
| Multiple pregnancy, n(%) | 55 (29.57) | 42 (26.25) | 13 (50.00) | χ² = 6.06 | **0.01** | |  |
| In vitro fertilization, n(%) | 30 (16.13) | 21 (13.12) | 9 (34.62) | χ² = 6.13 | **0.01** | |  |
| Meconium-stained amniotic fluid, n(%) | 11 (5.91) | 8 (5.00) | 3 (11.54) | χ² = 0.74 | 0.39 | |  |
| Premature rupture of membranes, n(%) | 64 (34.41) | 49 (30.62) | 15 (57.69) | χ² = 7.26 | **0.01** | |  |
| Antenatal corticosteroids, n(%) | 70 (37.63) | 54 (33.75) | 16 (61.54) | χ² = 7.36 | **0.01** | |  |
| Prenatal antibiotics exposure, n(%) | 33 (17.74) | 30 (18.75) | 3 (11.54) | χ² = 0.38 | 0.54 | |  |
| **Pre-NEC Clinical Characteristics** | | | | | | |  |
| Atrial septal defect, n(%) | 101 (54.30) | 88 (55.00) | 13 (50.00) | χ² = 0.23 | 0.64 | |  |
| Ventricular septal defect, n(%) | 12 (6.45) | 9 (5.62) | 3 (11.54) | χ² = 0.50 | 0.48 | |  |
| Patent ductus arteriosus, n(%) | 36 (19.35) | 30 (18.75) | 6 (23.08) | χ² = 0.27 | 0.60 | |  |
| Bronchopulmonary dysplasia, n(%) | 11 (5.91) | 8 (5.00) | 3 (11.54) | χ² = 0.74 | 0.39 | |  |
| Red blood cell transfusion, n(%) | 39 (20.97) | 35 (21.88) | 4 (15.38) | χ² = 0.57 | 0.45 | |  |
| Exclusive Breastfeeding, n(%) | 37 (19.89) | 32 (20.00) | 5 (19.23) | χ² = 0.01 | 0.93 | |  |
| Antibiotic Use, n(%) | 166 (89.25) | 143 (89.38) | 23 (88.46) | χ² = 0.00 | 1.00 | |  |
| **Blood Tests at the Time of Portal Venous Gas** | | | | | | |  |
| Platelet-to-C-reactive protein ratio  (×10^9^/mg), M(Q_1_,Q_3_) | 6.36 (3.43, 11.71) | 7.20 (4.35, 12.65) | 1.63 (0.75, 4.44) | Z = -4.97 | **< 0.01** | |  |
| **Abbreviations: PVG= portal venous gas** | | | | | | |  |
| t: t-test, Z: Mann-Whitney test, χ²: Chi-square test, -: Fisher exact | | | | | | |  |
| SD: standard deviation, M: Median, Q_1_: 1st Quartile, Q_3_: 3rd Quartile | | | | | | |  |
